# Paleopathological Evidence and Detection of *Mycobacterium leprae* DNA from Archaeological Skeletal Remains of *Nabe-kaburi* (Head-Covered with Iron Pots) Burials in Japan

**DOI:** 10.1371/journal.pone.0088356

**Published:** 2014-02-07

**Authors:** Koichi Suzuki, Aiko Saso, Keigo Hoshino, Junya Sakurai, Kazunari Tanigawa, Yuqian Luo, Yuko Ishido, Shuichi Mori, Kazuaki Hirata, Norihisa Ishii

**Affiliations:** 1 Leprosy Research Center, National Institute of Infectious Diseases, Tokyo, Japan; 2 The University Museum, The University of Tokyo, Tokyo, Japan; 3 Department of Biological Science, Graduate School of Science, The University of Tokyo, Tokyo, Japan; 4 Department of Anatomy, St. Marianna University School of Medicine, Kawasaki, Japan; 5 Faculty of Policy Management, Shobi University, Kawagoe, Japan; National Institute for Agriculture and Veterinary Research, IP (INIAV, I.P.), Portugal

## Abstract

The *Nabe-kaburi* is a unique burial method, the purpose of which is shrouded in mystery. The burials were performed during the 15^th^ to 18^th^ centuries in eastern Japan, and involved covering the heads of the deceased with iron pots or mortars. The identification of leprosy-specific osteological lesions among some of the excavated remains has led to the suggestion that *Nabe-kaburi* burials were a reflection of the social stigma against certain infectious diseases, such as leprosy, tuberculosis or syphilis. However, molecular evidence for the presence of disease has been lacking. The goal of this study was to detect *Mycobacterium leprae* (*M. leprae*) DNA in archaeological human skeletal remains from *Nabe-kaburi* burials. The paleopathological data from three *Nabe-kaburi* burials were re-evaluated before small samples were taken from affected and control areas. DNA was extracted and used as a template to target the *M. leprae*-specific DNA using a combination of whole genome amplification, PCR analysis and DNA sequencing. *M. leprae* DNA fragments were detected in the two sets of skeletal remains that had also shown paleopathological evidence of leprosy. These findings provide definitive evidence that some of the *Nabe-kaburi* burials were performed for people affected by leprosy. Demonstration of the presence of *M. leprae* DNA, combined with archeological and anthropological examinations, will aid in solving the mystery of why *Nabe-kaburi* burials were performed in medieval Japan.

## Introduction

The *Nabe-kaburi* burial was performed during the 15^th^ to 18^th^ centuries in the eastern region of Japan. In Japanese, “*nabe”* means pot and “*kaburi*” means to put on. Thus, the deceased were buried with an iron pot or mortar covering their heads. To date, a total of 105 *Nabe-kaburi* burials have been excavated in Japan [1].

There are two main theories in Japanese folklore as to why the deceased would be buried with iron pots on their heads [2], [3]. One is that the iron pots were worn to symbolically “block” spreading particular diseases, such as leprosy, tuberculosis or syphilis, which plagued the deceased when they were alive. The second reason is rather unique and somewhat humorous: *Nabe-kaburi* burials were performed for someone who died during the “Bon” period in Japan. The “Bon” is the Japanese ritual ceremony to welcome the souls of ancestors back from heaven during a 3-day period each summer. Since dying during the festival was considered imprudent, the ancestors beat the head of a descendant when they encountered each other on the way to and from the next world. Therefore, relatives of the descendant might have been trying to protect the head of the deceased at burial.

Such speculative stories serve to make the *Nabe-kaburi* burial appear more and more mysterious. However, a commonality between these burials and those of leprosy patients was noted as early as the initial study of the *Nabe-kaburi* burials more than 100 years ago. In addition, paleopathological examination of some cases revealed leprosy-specific skeletal changes in excavated specimens. During a time when there was no effective treatment, leprosy would have gradually spread over the entire body and caused specific osteological deformations in the nasal aperture, anterior nasal spine and alveolar process on the premaxilla, cortical areas of the tibia and fibula, distal ends of the metatarsals and diaphyses of the phalanges that may have included both direct and reactive changes [4]–[10].

**Figure 1:**
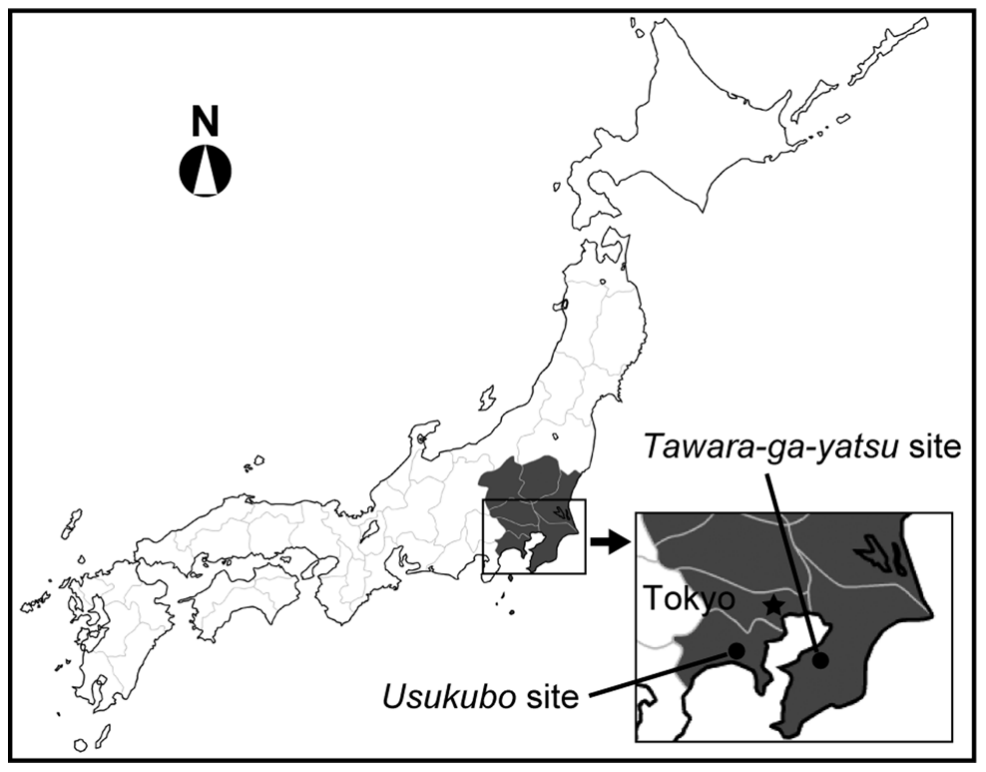
Location of the *Tawara-ga-yatsu* and the *Usukubo* sites in Japan. Shaded area denotes the *Kanto* area. Inset illustrates the locations in relation to Tokyo.

To date, 105 *Nabe-kaburi* burials have been found in Japan (Table 1). The oldest were buried in the 15^th^ to 16^th^ centuries, while the most recent have been dated to the 19^th^ century. About half were excavated from the *Kanto* region of Japan (Figure 1 shaded area). Most others were found in Northeast Japan, with only a few in Midland and Southwest Japan, including *Nagoya*, *Kyoto* and *Osaka*, which were already large cities at that time. Not all of the burial remains have had osteological and paleopathological evaluations, probably because skeletal preservation was poor and trained anthropologists were not always available. According to excavation reports released by the local governments, age was assessed in 25 cases. Age estimates of the excavated remains vary widely, ranging from approximately 10 years to greater than 60 years, but middle age was the most common. Fifteen cases were assessed to be male and seven to be female. Various types of pots were used to cover the heads, including iron pots, earthenware pots and mortars; however, iron pots were most frequently used (Table 2). The fact that such valuable items were used to cover the heads of the deceased suggests a particular importance of this burial style. Paleopathological examinations have been performed for 21 of the 105 burials. Five (24%) displayed osteological signs of leprosy, the most frequently observed of the disorders that were diagnosed based on osteological changes (Table 3). Based on folklore, tuberculosis is also thought to be a basis for *Nabe-kaburi* burials. However, to date, tuberculous lesions have not been found in the skeletal remains that have been subjected to paleopathological evaluation.

**Table 1:** Chronological and geographical distribution of *Nabe-kaburi* burials in Japan.

Age of burials	*Kanto* area^a^	Northeast Japan	Midland and Southwest Japan	Total
15^th^ –16^th^ century	9	11	2	22
16^th^ –18^th^ century	35	22	1	58
19^th^ century	−	2	−	2
Undetermined	7	14	2	23
Total	51	49	5	105

a Shaded area in Figure 1.

**Table 2:** Types of pots excavated from the *Nabe-kaburi* burials in each region.

Types	*Kanto* area^a^	Northeast Japan	Midland and Southwest Japan	Total
Iron pot with inside loop	7	15	−	22
Earthenware pot with inside loop	4	−	1	5
Iron/copper pot with lifted handles	9	8	1	18
Iron pot with pouring lip	18	9	−	27
Iron pot	4	2	1	7
Mortar	6	5		11
Others	3	2	2	7
Undetermined	4	8	−	12
Total	55	49	5	109^b^

a Shown as a shaded area in Figure 1.

b Total number of pots is greater than the number of *Nabe-kaburi* burials, because some remains were covered with more than two pots.

**Table 3:** Paleopathological lesions found in the skeletal remains excavated from the *Nabe-kaburi* burials.

Paleopathological diagnosis	Number of cases
Leprosy	5
Syphilis	3
Inflammatory lesions on the nose	1
Skeletal abnormality	1
No lesions	11
Total	21

In many societies, public stigmatization and exclusion coexist. Leprosy-associated deformities have been responsible for such social stigmatization and discrimination, and in some countries, the stigma is promoted by legislation against patients [10]. This commonality between *Nabe-kaburi* and leprosy burials led to speculation that *Nabe-kaburi* burials could to some extent reflect the discrimination against leprosy during that time period [2], [11]. Since 1980, with more excavations and archaeological findings (i.e., the grave goods, the age of the burials and the excavated human skeletal remains), the significance of the *Nabe-kaburi* burial has become an object of discussion not only in folklore but also in Early Modern archeology. It is believed that study of the *Nabe-kaburi* burials could reveal the reality of social discrimination against particular diseases in a village from the late Middle Ages to the Early Modern period in Japan. Therefore, confirmation that those skeletal remains showing osteological signs of leprosy were actually infected with *M. leprae* while alive became very important.

In the field of palaeomicrobiology, DNA from pathogenic microorganisms can be detected from excavated ancient human skeletons using the polymerase chain reaction (PCR) [12]. PCR is a powerful molecular tool for the discipline of palaeomicrobiology, diagnosing infectious diseases in ancient remains to demonstrate the distribution, spread and genetic evolution of the pathogens [13]–[15]. It can also be used for the genome-wide comparison of past and modern bacteria when DNA is well preserved [16].

*Mycobacterium tuberculosis* (*M. tuberculosis*) DNA was first detected in an ancient human skeleton using PCR in 1993 [17]. The next year, *M. leprae* DNA was detected in archaeological skeletal remains [18], which has been followed by several other reports [19]–[25]. We have recently reported detection of *M. leprae* DNA in excavated human skeletal remnants from Japan where preservation of ancient buried skeletons is poor due to humidity and acidic soil from volcanic activity [9]. Maximum sensitivity was achieved with a method that combined whole genome amplification (WGA) and PCR analysis (WGA-PCR) and DNA sequencing [9].

In the present study, we examined three sets of skeletal remains from *Nabe-kaburi* burials. Although paleopathological evidence was briefly documented in reports by local governments, we examined skeletons and re-evaluated the paleopathological findings. We then analyzed small amounts of these samples for the presence of *M. leprae* DNA using WGA-PCR and DNA sequencing.

## Materials and Methods

### Archeological Sites and Graves of *Nabe-kaburi* Excavations

This study used archaeological skeletal remains excavated from two sites in the *Kanto* area of Japan (Figure 1): the *Tawara-ga-yatsu* and *Usukubo* sites. Below is a brief summary of the excavation sites and graves described in excavation reports obtained from the local governments in Japanese.

**Figure 2:**
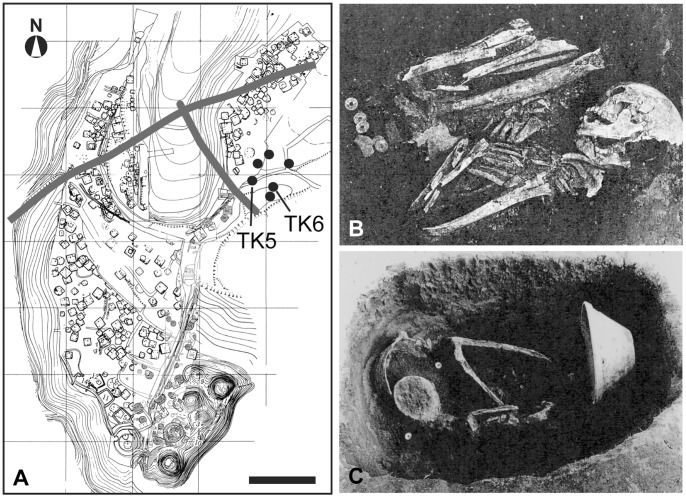
Geographical representation of the *Tawara-ga-yatsu* site. (A) Map of the *kobama* ruins including the *Tawara-ga-yatsu* site where TK5 and TK6 were excavated. Thick lines indicate roads that cross the site. TK5 (B) and TK6 (C) grave pits at excavation. Bar = 50 m.

The *Tawara-ga-yatsu* site is part of the *Kobama* ruins spreading on hills along the coastline of Tokyo Bay located on the *Boso* Peninsula in Chiba prefecture [26], [27]. Perpendicular to the structural remains of roads traversing from south to north are groove-shaped structural remains extending from east to west (Figure 2A). Six grave pits were found annularly arranged on the side of these structural remains. The *Nabe-kaburi* burials were excavated from two of these gravel pits (TK5 and TK6) (Figure 2A). The head of the remains in TK5 was covered with two iron pots and one mortar. The grave goods consisted of *Kan-ei-tsuho* (a metallic currency used in the *Edo* period) and three glass beads (Figure 2B). The corpse in TK6 was covered with one iron pot (head) and one mortar (feet), with grave goods of five *Kan-ei-tsuho* (Figure 2C). Based on the shape of the pots and the presence of the metallic currency, both burials were assessed to be from the early 18^th^ century. TK5 was assessed to have been a middle-aged male, while TK6 was assessed to have been a male in his late 20 s. It was estimated that the rounding deformation seen in the lateral and inferior edges of his nasal aperture resulted from leprosy [28].

**Figure 3:**
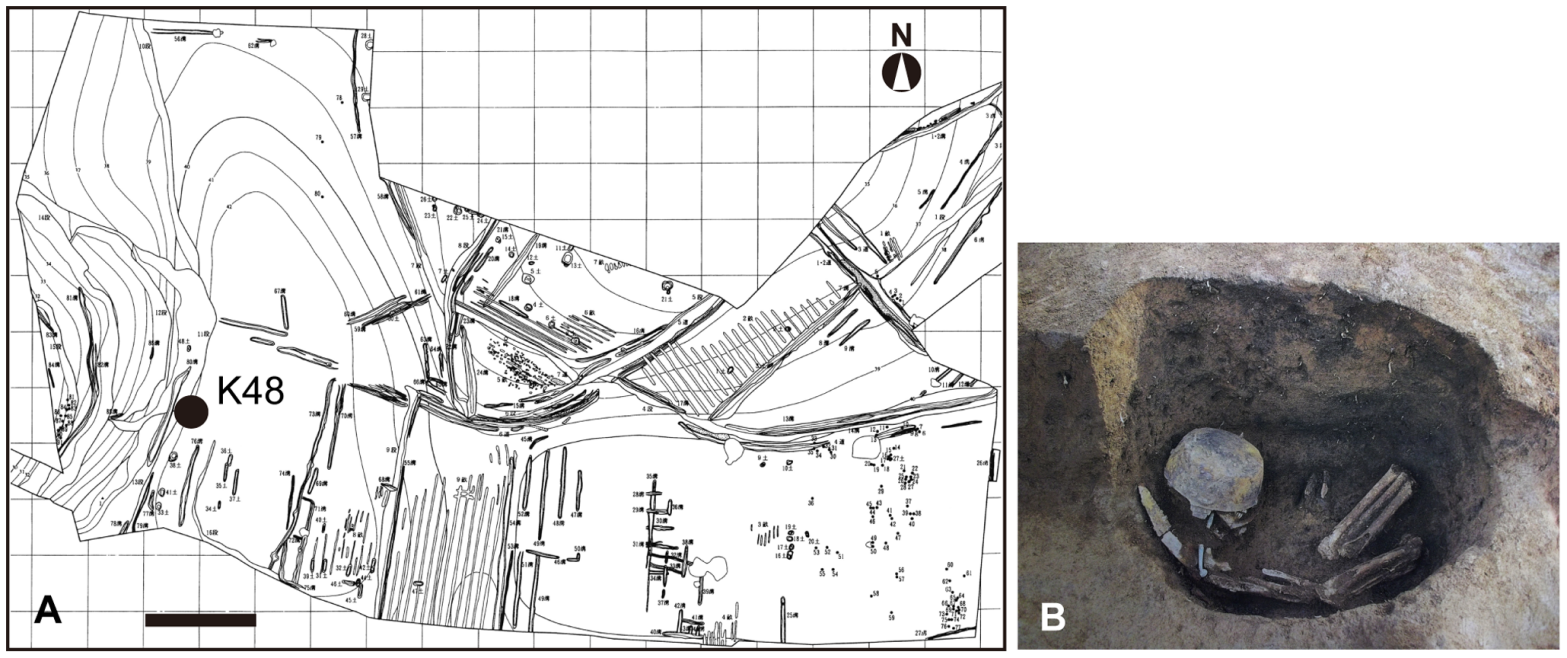
Geographical representation of the *Usukubo* site. (A) Map of the *Usukubo* site where K48 was excavated. (B) K48 grave pit at excavation. Bar = 20 m.

The *Usukubo* site refers to ruins located on the lingulate plateau of the plains facing *Sagami* Bay in Kanagawa prefecture, where large-scale colonies dated to 5,000 B.C. (the *Jomon* period) have been excavated [29] (Figure 3A). A bent human skeleton with an iron pot covering the head was excavated from an 18^th^ century grave pit (K48) (Figure 3B). K48 is located near the border of the village next to a ridge traversing the center of the plateau from east to west through farmland. A smaller iron pot, a tobacco pipe and a knife-like iron tablet were also in the pit. The estimated date of this burial is early to middle 18^th^ century, based on the shape of the tobacco pipe and the properties of the covered soil. The human skeleton excavated from K48 was assessed to be a middle-aged man with collapse of the central facial cranium and bone hypertrophy in most of his limb bones, both of which are suggestive of leprosy.

### Sampling of Skeletal Remains

Sampling from three skeletal remains was performed at the Shiomi Storehouse of Board of Education, Kisarazu City, 7-3-7 Shiomi, Kisarazu 292-0834, Japan (for TK5 and TK6) and the National Museum of Nature and Science in Tokyo, 3-23-1 Hyakunincho, Shinjuku 169-0073, Japan (for K48), where no other leprous materials were stored. Skeletal samples were taken from the affected lesions and from bones with no remarkable changes, as summarized in Table 4, using a small rotating electric saw. Sterile materials were used for sampling to avoid possible contamination. Ethical approval to work with the material was obtained from the review board at the National Institute of Infectious Diseases, Japan. Permission to obtain the sample materials was granted by the Board of Education of Kisarazu City, Kisarazu, Japan and the National Museum of Nature and Science, Tokyo, Japan. All necessary permits were obtained for the described study, which complied with all relevant regulations.

**Table 4:** Skeletal samples analyzed for the presence of *M. leprae* DNA.

Sample No.	Material reference	Sampling site	Paleopathological evidence	Sample weight (mg)	*M. leprae* DNA
1	TK5	Femur	N.R.	80.1	−
2	TK5	Inner surface of nasal cavity, left	N.R.	41.9	−
3	TK6	Astragalus, left	Erosion/atrophy	90.0	−
4	TK6	Lower 2nd molar root, right	N.R.	87.7	−
5	TK6	Inner surface of nasal cavity, right	Erosion/atrophy	52.2	+
6	TK6	Maxillary palate, right	Erosion/atrophy	87.5	−
7	K48	Nasal septum	Erosion/atrophy	139.0	−
8	K48	Inferior nasal concha, right	Erosion/atrophy	183.0	−
9	K48	Fibular diaphysis	Periostitis	184.2	−
10	K48	Inner surface of nasal cavity, right	Erosion/atrophy	48.0	+
11	K48	Upper 3rd molar root, right	N.R.	252.6	−

N.R.: No remarkable change.

### DNA Extraction, whole Genome Amplification (WGA), Polymerase Chain Reaction (PCR), and DNA Sequencing

Genomic DNA was purified using QIAamp DNA Micro Kit (Qiagen, Valencia, CA), and uniformly amplified using the GenomePlex Whole Genome Amplification Kit (Sigma, St. Louis, MO) as reported previously [9]. Eight *M. leprae*-specific primer pairs were used to amplify 1) coding regions: *ML2496c* (*dnaK*; *hsp-70*), *ML2205c* (*purM*) and *ML1309* (*hisE*); 2) pseudogenes: *ML0434* (*scoA*) and *ML0794c* (REP-family protein); and 3) non-coding regions: Nc1593211, Nc2551060 and Nc2664658, where the numbers denote coordinate positions within the *M. leprae* genome [9]. These primers were chosen to generate shorter amplicon sizes, which produce more efficient amplification of degraded samples. Nested (for *ML2496c*) and conventional PCR (remaining regions) were performed using the PCR Thermal Cycler DICE (TaKaRa) [9]. The PCR products were analyzed using 2.0% agarose gel electrophoresis. For DNA sequencing, specific PCR products were cut from an agarose gel and DNA was extracted using the MinElute Gel Extraction Kit (Qiagen). DNA sequencing was performed and analyzed using the ABI PRISM 310 Genetic Analyzer and GeneScan Collection software (Applied Biosystems) [9].

All experiments were performed in a biosafety level 2 (BSL2) facility with secure access; only trained and authorized persons are allowed to enter. To work in a BSL2 laboratory, one must take a one-day lecture course on basic biosafety, pass a multiple choice exam, and have a minimum of 30 hours (depending on previous experience) of actual training in the laboratory under strict supervision of the laboratory manager. Although *M. leprae*, or its DNA purified from patients or from nude mice foot pads, is handled daily in the laboratory for diagnoses or other basic research, it is handled in a Class II biological safety cabinet in which both inward and outward (exhaust) air are passed through HEPA filters to avoid cross contamination. Only sterile disposable test tubes and other materials such as disposable gloves, masks and labware are used. All waste is packaged in a plastic bag for biohazardous materials, autoclaved for sterilization and disposed of as medical refuse to avoid contamination. Although the experimental practices of the laboratory may differ somewhat from the guidelines proposed for the detection of aDNA [12], [30], [31], the laboratory can reliably and consistently detect minute amounts of *M. leprae* DNA from both patient samples and archeological specimens.

## Results

### Paleopathological Evaluation and Diagnosis

#### TK5 burial

**Figure 4:**
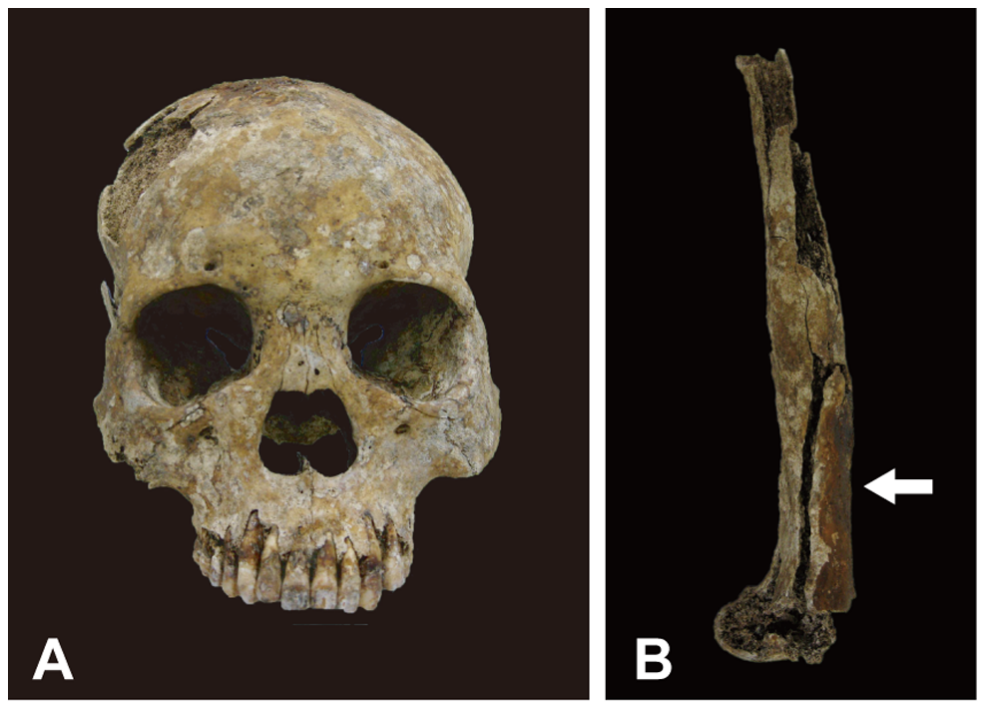
Macroscopic view of the osteological lesions in the skeletal remains of TK5. (A) Frontal view of the skull with no paleopathological signs. The area from the glabella to the nasion and inion was in plane. No periostitis-caused osteological lesions were observed in the nasal bone or on the edges of the nasal aperture. (B) View of diaphysis of the femur. The arrow indicates extensive hypertrophy in the lower bone.

The skull of TK5 was almost intact and in a good state of preservation (Figure 4A), while most parts of the trunk bones were not preserved. The distal ends of the limb bones were also absent, leaving only the diaphysis. No paleopathological signs were observed in the skull (Figure 4A). Although the area from the glabella to the nasion and inion was in a plane and the profile appeared somewhat unique, no periostitis-induced osteological lesions were observed in the nasal bone or on the edges of the nasal aperture. Extensive hypertrophy was seen in the lower part of diaphysis of the femur, indicating that TK5 was suffering from either periostitis or osteomyelitis (Figure 4B). Although TK5 was excavated from a *Nabe-kaburi* burial and initially suspected of having syphilis of the bone (based on an excavation report provided by the local government), the lesions were limited to the diaphysis of femurs without any syphilis-specific signs. Therefore, it was more likely that the lesions were caused by extensive trauma- or infection-induced periostitis or osteomyelitis rather than syphilis [4], [8], [12].

One small piece of bone fragment with osteological lesions was taken from the femur for analysis, and another sample with no osteological lesions was taken as a control from the inner surface of the nasal cavity on the left frontal process of maxilla.

#### TK6 burial

**Figure 5:**
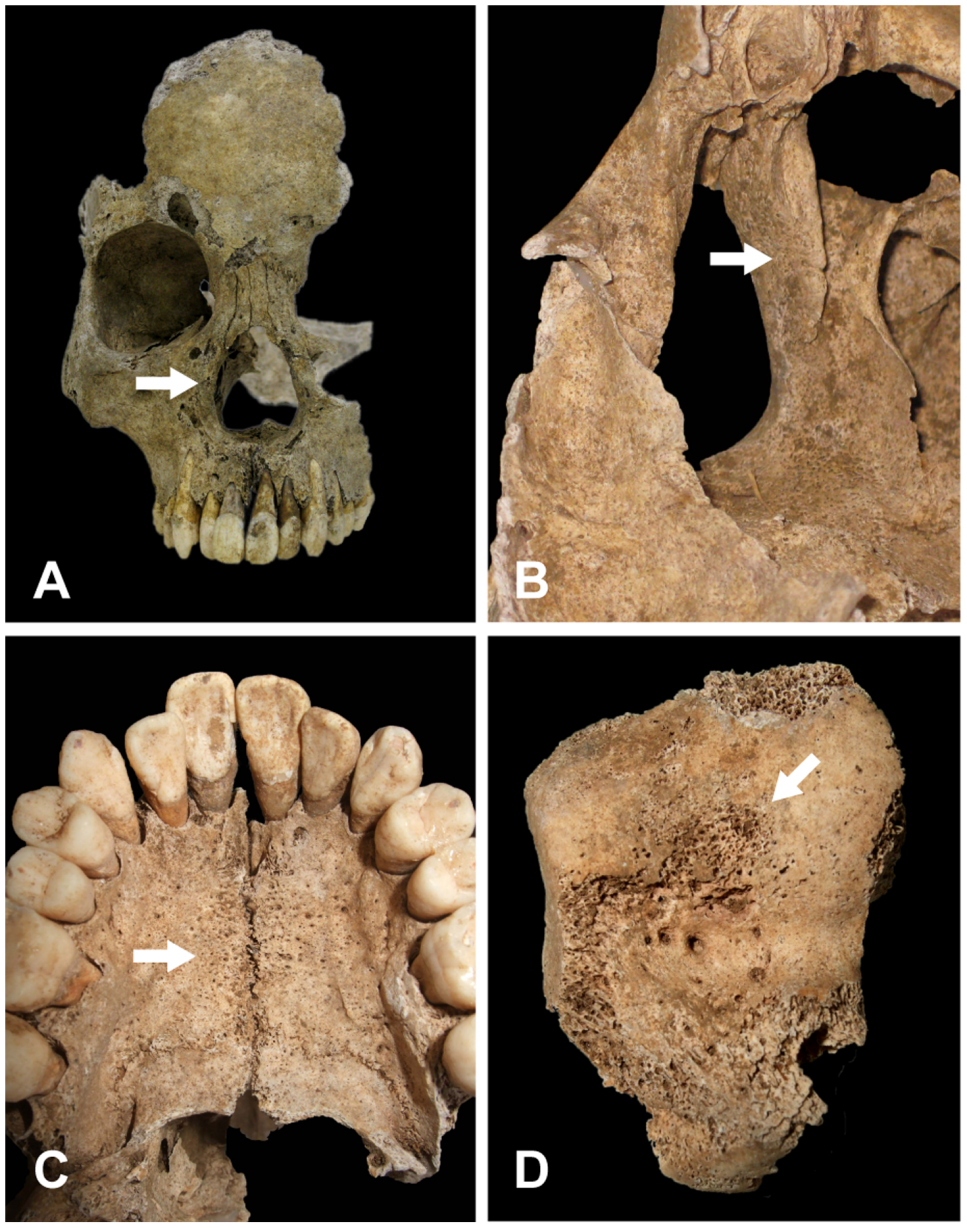
Macroscopic view of the osteological lesions in the skeletal remains of TK6. (A) Frontal view of the skull. Two thirds of the right facial cranium and the mandible remained, while the cerebral cranium was missing. The arrow indicates symmetrical hypertrophy in the cortical bone around the edges of the nasal aperture, which was especially remarkable in the area between the lateral edge to the inferior edge. (B) Closer view of the rough and corrugated inner surface of the nasal cavity (arrow), indicating bone absorption and remodeling in the palatine process of maxilla, the frontal process of maxilla and the inferior nasal concha. (C) Closer view of the maxillary palate. Densely packed small holes (arrow) around the palatine suture on the surface of palatine process of maxilla are indicative of osteoporosis. (D) Closer view of the talus. Lesions and depressions (arrow) in the area between the frontal middle trochlea and talar neck, adjacent to the inner surface of squatting facets on talus, exhibit a porous surface, which is also indicative of osteoporosis.

Excavation of TK6 revealed that skeletal preservation was very poor. Only two thirds of the right facial cranium and the mandible remained, and the cerebral cranium was missing. Bones that were present included the right femur, left talus and several pieces of bone from the tibia and sacrum. Paleopathological signs of leprosy were observed around the nasal aperture in the skull (Figure 5A). The edges of the nasal aperture were rounded, and the cortical bone around the edges displayed symmetrical hypertrophy, especially remarkable in the area between the lateral edge to the inferior edge. The rough and corrugated inner surface of the nasal cavity indicated bone absorption and remodeling in the palatine process of maxilla, the frontal process of maxilla and the inferior nasal concha, which were likely caused by periostitis (Figure 5B). Densely packed small holes, indicative of osteoporosis, were observed around the palatine suture on the surface of the palatine process of maxilla (Figure 5C). Moreover, atrophy of the nasal spine, atrophy of the alveolar bone around the prosthion and inflammatory changes in the surface of the cortical bone on the anterior teeth were confirmed. These osteological lesions limited to the anterior nasal spine, nasal aperture and nasal cavity are believed to be specific to leprosy [32]. Probable inflammatory depressions were noted in the area between the frontal middle trochlea and the talar neck (Figure 5D). They were adjacent to the inner surface of squatting facets on the talus, exhibiting a porous surface, which is again indicative of osteoporosis or non-specific inflammation due to a mixed infection associated with nerve damage and loss of sensation. This is a rather unique lesion as leprosy-related osteological changes are rarely reported in the talus [4], [8], [12], [33].

Three samples with osteological lesions were taken from the inner surface of the nasal cavity on the right frontal process of maxilla, right maxillary palate and the center of the left talar neck. A control sample with no osteological lesions was taken from the lower second molar root on the right mandible.

#### K48 burial

**Figure 6:**
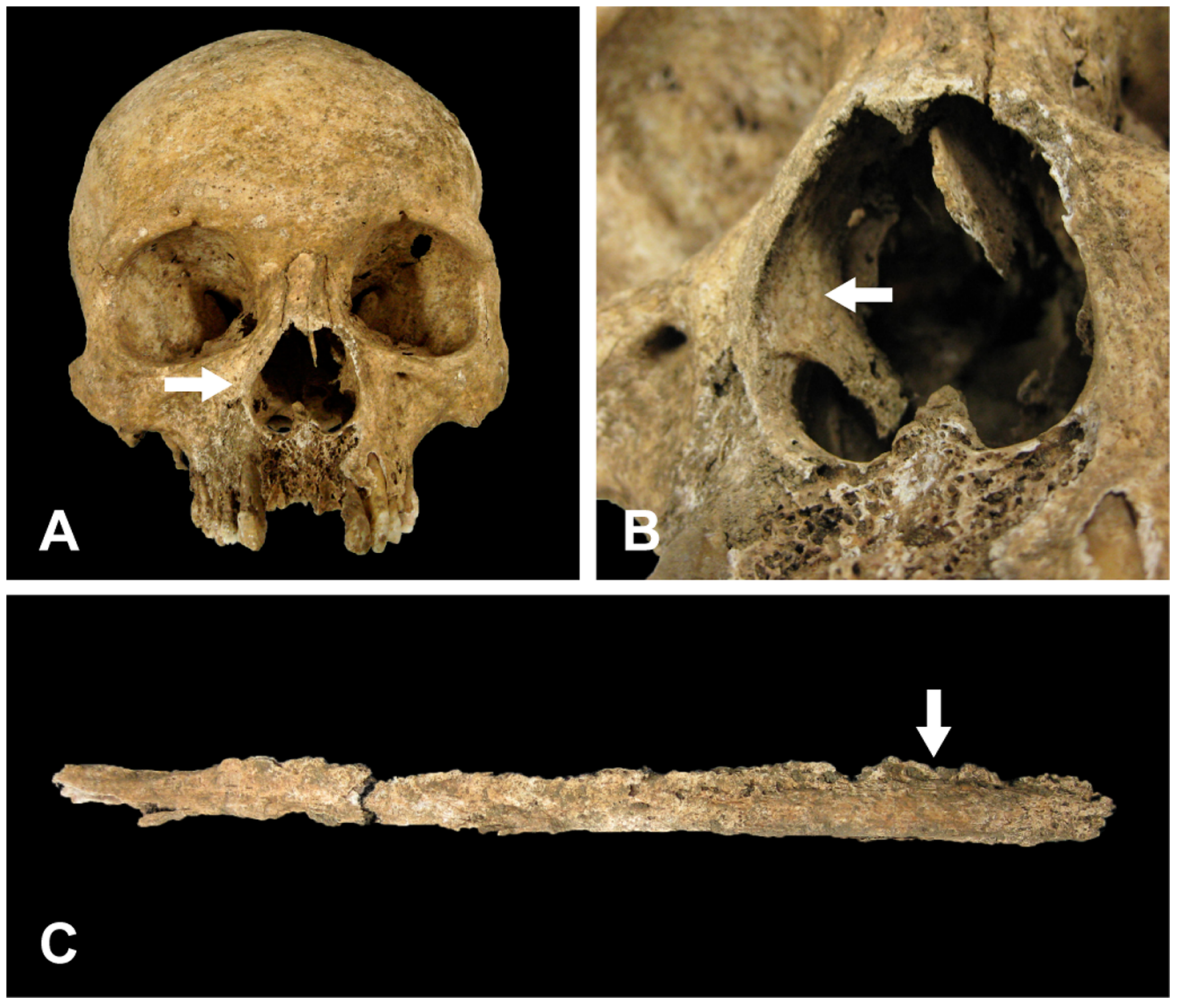
Macroscopic view of the osteological lesions in the skeletal remains of K48. (A) Frontal view of the skull. Porotic hyperosteosis is evident in the area around the rounded edges of the nasal aperture, especially remarkable on the surface of the frontal process of maxilla (arrow). (B) Closer view of the nasal aperture. The inner surface of nasal cavity lacked signs of periostitis-related hyperosteosis (arrow). (C) The fibula showed advanced hypertrophy and generalized swelling with the typical leprosy-specific feature of “vascular grooves” (arrow).

Bone erosion with porotic hyperosteosis was observed in the area around the rounded edges of the nasal aperture, and was especially remarkable on the surface of the frontal process of maxilla (Figure 6A). Periostitis-related hyperosteosis was not seen on the inner surface of the nasal cavity or in the inferior nasal concha (Figure 6B). It has been reported that the anterior teeth are susceptible to leprosy, thus the loss of teeth between the central incisor and the lateral incisor occurs in advanced leprosy cases [4], [7]. There was severe damage to the alveoli of the anterior teeth of maxilla and exposure of cancellous bone. Although the trace of the alveolar foramina of the right first incisor was not found, the absence of anterior teeth may not be due to any kind of diseases as there were no specific signs of deformation or bone absorption on the surrounding bone tissues. The limb bones of K48 were very fragile and in a poor state of preservation. Nevertheless, significant hypertrophy was clearly seen in the fibula with “vascular grooves” that are characteristic of leprosy (Figure 6C).

Four samples with osteological lesions were taken from the nasal septum, right inferior nasal concha, inner surface of the right nasal cavity and diaphysis of the right fibula. A sample from the upper third molar root on the right maxilla was taken as a control.

### Detection of *M. leprae* DNA by WGA-PCR and DNA Sequencing

**Figure 7:**
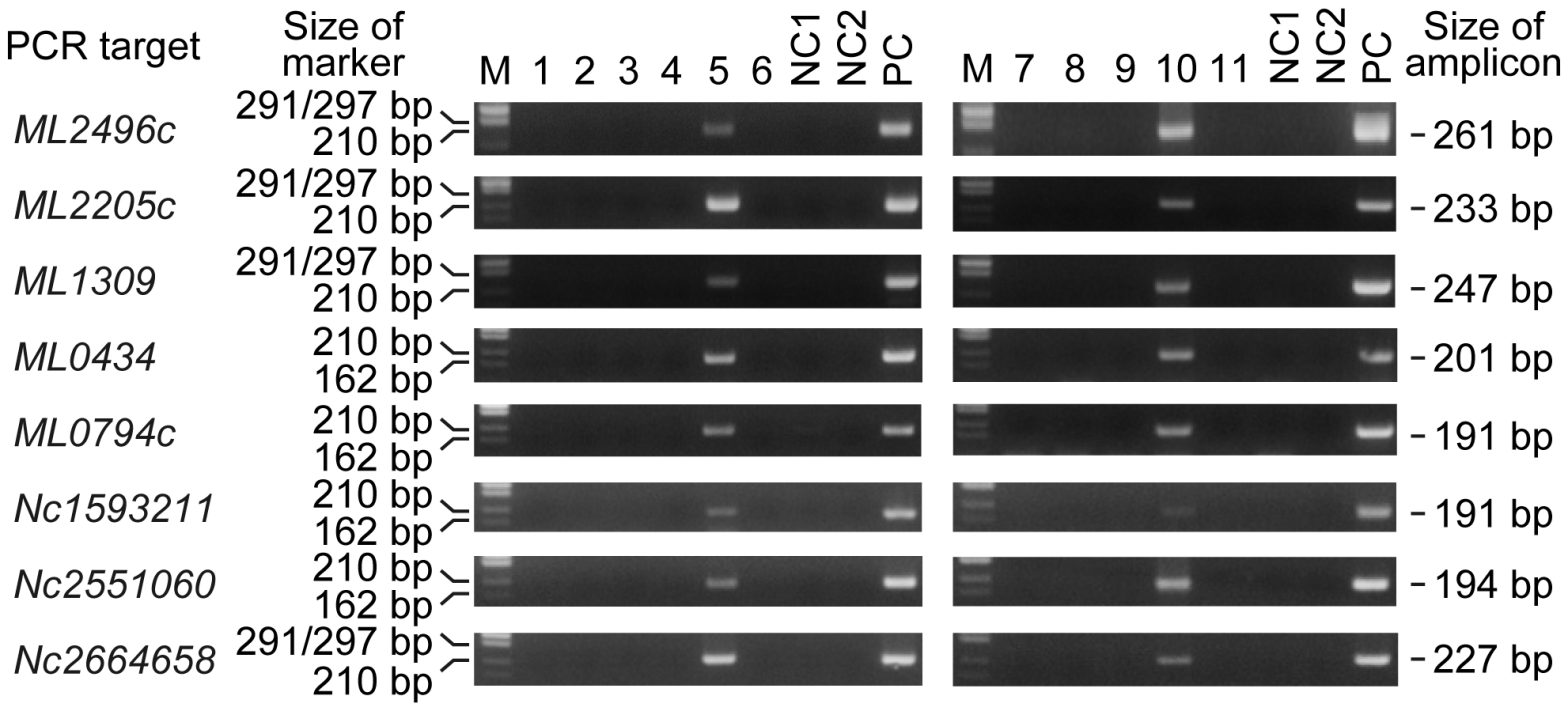
Detection of *M. leprae* DNA from skeletal samples. PCR analysis was performed using *M. leprae*-specific primers to detect three coding genes, two pseudogenes and three non-coding regions. Coding genes: *ML2496c* (*dnaK*; *hsp-70*), *ML2205c* (*purM*), *ML1309* (*hisE*); pseudogenes: (*ML0434* (*scoA*) and *ML0794c* (REP-family protein); and non-coding regions: Nc1593211, Nc2551060 and Nc2664658. Numbers denote coordinate positions within the *M. leprae* genome. PCR products were evaluated using 2% agarose gel electrophoresis. M: DNA size marker (φX174 *Hinc* II digest, Takara, Otsu, Japan). NC1: a negative control for DNA purification in which DNase/RNase-free water was used as the sample during the process of DNA extraction. NC2: a negative control for PCR in which DNase/RNase-free water was used instead of a DNA sample. PC: positive control DNA from the *Thai 53* strain of *M. leprae*.

DNA was purified from 11 skeletal samples from three individuals (TK5, TK6 and K48) excavated from two different sites as shown in Table 4. The purified genomic DNA was then used as a template for PCR amplification of *M. leprae* genes, pseudogenes and non-coding regions using WGA-PCR, a method with a high sensitivity for the detection of *M. leprae* DNA from ancient samples [9]. Among seven samples taken from paleopathological leprosy lesions (No. 3, 5 and 6 from TK6 and No. 7–10 from K48; see Table 4), *M. leprae*-specific DNA fragments were amplified in two: sample No. 5 (inner surface of the right nasal cavity of TK6) and sample No. 10 (inner surface of the right nasal cavity of K48) (Figure 7 and Table 4). Control samples taken from TK6 and K48, and all of the samples from TK5, a *Nabe-kaburi* burial case without paleopathological evidence of leprosy, were negative for PCR detection. The specificity of these PCR amplifications was confirmed by DNA sequencing of the PCR products purified from agarose gel (Figures S1A and S1B). A Basic Local Alignment Search Tool (BLAST) search of the two DNA sequences revealed a 100% match to the reported *M. leprae* sequence for the *ML2496c* gene (Figure S1C). These results clearly confirmed that *M. leprae* infection was present in the paleopathological lesions in samples from the TK6 and K48 *Nabe-kaburi* burials.

## Discussion

The *Nabe-kaburi* burial is a ritual of interment of the deceased with an iron pot covering his/her head. Although paleopathological characteristics of leprosy have been noted in some cases, there was no definitive evidence to prove that the skeletal remains belonged to persons who died with leprosy. In this study, we took small amounts of samples from skeletal remains having lesions that were thought to be related to leprosy. Although preservation of the bones was rather poor, highly sensitive WGA-PCR analysis and subsequent DNA sequencing clearly demonstrated that two cases were infected with *M. leprae*: one is TK6, excavated from the *Tawara-ga-yatsu* site, and the other is K48, excavated from the *Usukubo* site. Thus, we have for the first time used molecular techniques to confirm that people with leprosy were buried in the *Nabe-kaburi* burials by using eight different PCR primers and a DNA sequence of one of the PCR products (*i.e. ML2496c*). Although three samples from different affected areas were taken from TK6 and four samples from K48, in each case only one sample taken from the inner surface of the nasal cavity was successfully amplified by WGA-PCR, suggesting that multiple sampling including the inner surface of the nasal cavity is necessary for detection of *M. leprae* DNA.

Contamination of DNA is a big concern in the fields of archeology and anthropology [12], [30], [31], especially when analyzing human DNA. Our laboratory is eligible to handle infectious agents as described in the Materials and Methods and is responsible for the molecular diagnosis of all the newly found leprosy cases in Japan. Although the experimental practices of our laboratory may differ somewhat from the guidelines proposed for the detection of aDNA, we follow strict guidelines for preventing contamination to ensure the correct molecular diagnosis in the laboratory. Therefore, we are confident that data from our laboratory is reliable enough to demonstrate minute amounts of *M. leprae* DNA, no matter whether samples are taken from current patients or from ancient skeletal remains.

The grave goods found in the *Nabe-kaburi* burials were similar to those in the common cemetery. Moreover, in some cases, but not in the present cases, devices have been found beneath the graves of the *Nabe-kaburi* burials, which is indicative of some sort of memorial events that took place for a certain period of time [34]. Since such ceremonies were performed to “decontaminate” the spirits of those with unusual causes of death, it is possible that people who died of not only specific diseases such as leprosy or syphilis, but also unfortunate accidents or incidents may likely have been interred in the *Nabe-kaburi* burials. In this sense, the *Nabe-kaburi* burial, rather than being a symbol of discrimination, could have served as salvation of the unfortunate souls.

The evidence confirmed by the present study also suggests a possibility that is different from discrimination: 1) in TK6 and K48, deceased persons were buried with some grave goods and coins, the latter believed to be needed as a passage fee when the deceased crosses a river to go to heaven; 2) valuable and probably very important items, (*e.g.,* iron pots) were buried with the diseased; and most importantly 3) both TK6 and K48 stayed alive long enough to have leprosy-specific lesions form on their skeletons, despite the fact that progressive lepromatous leprosy cases usually have severe deformities, such as losing fingers, severe oral and nasal defects, and possible blindness or lagophthalmos due to inflammation [10]. This evidence suggests that these leprosy patients were cared for by someone, they were provided with food and other daily cares for a long period of time so that they were able to stay alive when the disease further progressed. When they died, they were buried in the same manner as other people in the village with only the exception of the iron pots covering their heads. Therefore, it must be considered that society took good care of leprosy patients, at least in the cases of TK6 and K48, but was afraid of transmission of the disease from the graves and tried to decontaminate the souls. It can be speculated that valuable iron pots were used as a kind of tribute.

At this stage, it is still too early to draw a solid conclusion about the significance of the *Nabe-kaburi* burials. However, in contrast to the paleopathological diagnosis based solely on macroscopic changes in skeletal remains, this study has for the first time made a definitive diagnosis of leprosy using molecular methods, thus providing undeniable evidence of advanced leprosy in two cases excavated from the *Nabe-kaburi* burials. These methods will be valuable tools in future attempts to demonstrate the presence of *M. tuberculosis* or *Treponema pallidum* DNA if samples exhibiting the typical osteological lesions of tuberculosis or syphilis are obtained. Traditional paleopathological findings combined with molecular analysis will no doubt provide more reliable and definitive evidence, thus promoting the study of the significance of the *Nabe-kaburi* burial.

## Supporting Information

Figure S1 **Sequencing of** ***M. leprae*** **DNA from skeletal samples.** Original chromatogram of DNA sequencing of sample No. 5 from TK6 (A) and sample No. 10 from K48 (B). (C) Partial DNA sequence of *M. leprae ML2496c* (*dnaK*; *hsp-70*) gene. Underlining denotes the sequence data showed a 100% match to the original sequence.
(TIF)
