# Supplementary figures and images for: Paleopathological Evidence and Detection of Mycobacterium leprae DNA from Archaeological Skeletal Remains of Nabe-kaburi (Head-Covered with Iron Pots) Burials in Japan
Source: PLoS One. 2014 Feb 7;9(2):e88356. doi: 10.1371/journal.pone.0088356 (PMC3917912; doi:10.1371/journal.pone.0088356)

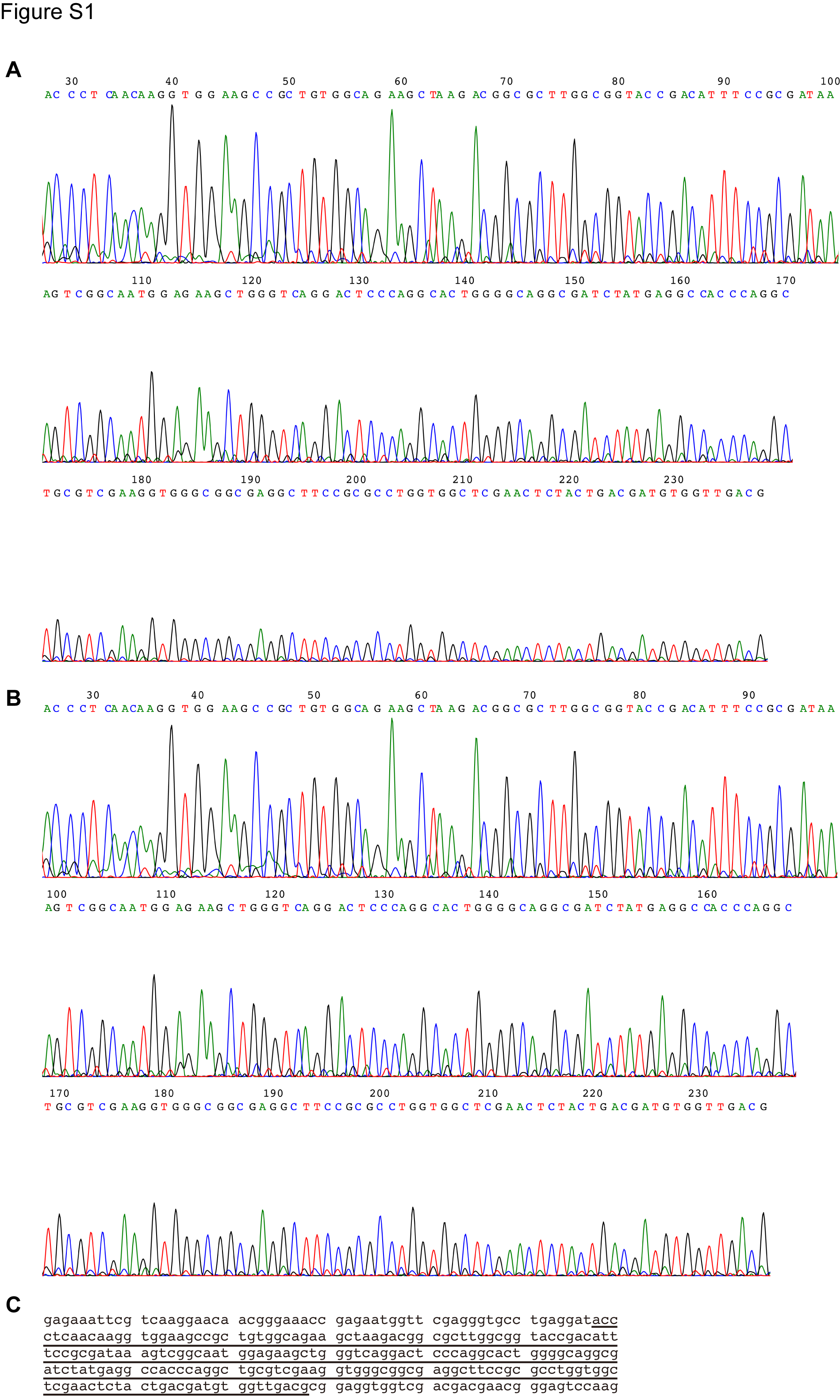

Supplement: Figure S1 — Sequencing of M. leprae DNA from skeletal samples. Original chromatogram of DNA sequencing of sample No. 5 from TK6 (A) and sample No. 10 from K48 (B). (C) Partial DNA sequence of M. leprae ML2496c (dnaK; hsp-70) gene. Underlining denotes the sequence data showed a 100% match to the original sequence. (TIF) [file pone.0088356.s001.tif]
